# Boosting the Electrostatic MEMS Converter Output Power by Applying Three Effective Performance-Enhancing Techniques

**DOI:** 10.3390/mi14020485

**Published:** 2023-02-19

**Authors:** Mona S. Salem, Abdelhalim Zekry, Mohamed Abouelatta, Ahmed Shaker, Marwa S. Salem, Christian Gontrand, Ahmed Saeed

**Affiliations:** 1 Electronics and Communications Engineering Department, Faculty of Engineering, Ain Shams University (ASU), Cairo 11566, Egypt; 2 Physics and Mathematics Engineering Department, Faculty of Engineering, Ain Shams University (ASU), Cairo 11566, Egypt; 3 Department of Computer Engineering, Computer Science and Engineering College, University of Ha’il, Ha’il 55211, Saudi Arabia; 4 National Institute of Applied Sciences of Lyon (INSA Lyon), 69621 Lyon, France; 5 IEP, INSA—Fès, Université Euro-Méditerranéenne de Fès, Fès 30120, Morocco; 6 Electrical Engineering Department, Faculty of Engineering and Technology, Future University in Egypt, New Cairo 11835, Egypt

**Keywords:** performance enhancement, MEMS converter, vibration, boosting the output power, COMSOL Multiphysics 5.4

## Abstract

This current study aims to enhance the electrostatic MEMS converter performance mainly by boosting its output power. Three different techniques are applied to accomplish such performance enhancement. Firstly, the power is boosted by scaling up the technology of the converter CMOS accompanied circuit, the power conditioning, and power controlling circuits, from 0.35 µm to 0.6 µm CMOS technology. As the converter area is in the range of mm^2^, there are no restrictions concerning the scaling up of the accompanied converter CMOS circuits. As a result, the maximum voltage of the system for harvesting energy, *V_max_*, which is the most effective system constraint that greatly affects the converter’s output power, increases from 8 V to 30 V. The output power of the designed and simulated converter based on the 0.6 µm technology increases from 2.1 mW to 4.5 mW. Secondly, the converter power increases by optimizing its technological parameters, the converter thickness and the converter finger width and length. Such optimization causes the converter output power to increase from 4.5 mW to 11.2 mW. Finally, the converter structure is optimized to maximize its finger length by using its wasted shuttle mass area which does not contribute to its capacitances and output power. The proposed structure increases the converter output power from 11.2 mW to 14.29 mW. Thus, the three applied performance enhancement techniques boosted the converter output power by 12.19 mW, which is a considerable enhancement in the converter performance. All simulations are carried out using COMSOL Multiphysics 5.4.

## 1. Introduction

Recent studies focus on developing low-power, portable, and remote devices. Such development contributes to replacing traditional energy sources with untraditional ones. Thus, it is important to harvest environmental energy because, in applications located in a non-reachable environment where maintenance costs are high, harvesting the energy from the environment becomes essential. Biomedical devices and remote area wireless sensors are some examples of applications that need energy harvesters [1,2,3,4]. Harvesting energy means converting environmental energy directly into electrical energy. Solar, thermal, vibration, and wind energy are examples of environmental energy [5,6,7,8]. Concerning the vibration energy, the MEMS harvester is normally used. It consists of a spring and a mass. It resonates at one or more of the input vibration frequencies. MEMS vibration harvesters have three types: electrostatic, electromagnetic, and piezoelectric harvesters [9,10,11,12,13]. Such types are based on the transfer mechanism. In an electromagnetic harvester, its coil resistance causes losses which are considered one of its main drawbacks. Additionally, the fabrication processes of this type of harvester are complex and its process compatibility is low [14]. For the electrostatic energy harvester, its fabrication processes are easy as it uses the micro-machining standard processes [15,16,17,18]. The required type of MEMS harvester is determined based on the required power density in a certain application. Electrostatic harvesters dominate piezoelectric when the accelerations are low. The reason is that its energy loss is low. At very high accelerations, the piezoelectric harvester performance is greatly degraded, which is caused because of its dielectric breakdown limit. At very high accelerations, the electrostatic harvesters are better. Thus, the optimal mechanism of transduction is based on the operating frequency, device size, and harvesting acceleration [19,20,21,22,23,24,25]. Electrostatic harvesters are based on the mechanism of capacitive sensing, which is considered the dominant mechanism for micro-machined applications. This is because of its compatibility with all the fabrication processes [26,27,28,29]. Recently, the enhancement of the electrostatic MEMS converter performance becomes important. The enhancement is mainly related to improving its output power. Until now, it still ranges from µW to a few mW [30,31,32,33]. Additionally, another research direction concerns the accompanied converter circuit. Such direction is necessary for treating the output power to be delivered to the load. [34,35,36,37].

In this current study, the main objective is to enhance the converter power based on investigating the main key performance factors which effectively affect the converter performance. Such main factors are the maximum voltage of the energy harvesting system, *V_max_*, the converter technological parameters, and the optimization of the converter structure. Our research group has previous efforts concerning the enhancement of the electrostatic MEMS converter output power. Its power was initially enhanced by depositing a high dielectric material, which is the tantalum pentoxide, Ta_2_O_5_, on its sidewall fingers [38]. The converter power increases to a few mWs, from 0.09 mW to 2.1 mW. Such values are considered a remarkable enhancement in comparison with recent studies [30,31,32]. In this paper, our main contribution is to boost the electrostatic MEMS converter output power by applying three effective performance enhancement techniques. Each applied technique aims to effectively enhance one of the main key performance factors which greatly improves the converter output power. The first technique is carried out by scaling up the technology of the converter CMOS circuit, the power conditioning, and power control circuits, from 0.35 µm to 0.6 µm CMOS technology. This scaling up increases the maximum voltage of the energy harvesting system, *V_max_*, from 8 V to 30 V. As *V_max_* is the most effective system constraint that greatly affects the converter output power [38], this output power is nearly doubled by applying this technique. Secondly, the converter output power is enhanced by optimizing its technological parameters, thickness, finger width, and length. Finally, the converter structure is optimized to overcome its wasted shuttle mass area. Thus, the converter finger length, which is the most effective technological parameter in enhancing its output power, is maximized.

The paper is arranged as follows. Section 2 illustrates the spring design which is responsible for the frequency tuning of the converter. In Section 3, the COMSOL Multiphysics 5.4 is calibrated by using a case study of 0.35 µm CMOS technology. Moreover, the required simulation results for calibrating the tool, including electric potential distribution, electric field distribution, the converter displacement, stress analysis, and the output power at different input voltage, are presented. In Section 4, a qualitative analysis of the three applied effective techniques used for enhancing the electrostatic MEMS converter performance based on its power equation is illustrated. In Section 5, the qualitative analysis is evaluated and verified using COMSOL Multiphysics 5.4. Finally, the conclusions and the important findings of this work, along with our future work, are offered in Section 6.

## 2. Electrostatic MEMS Converter Spring Design

In our previous research, we built up a proposed electrostatic MEMS converter behavioral circuit model. The published model represented the converter comb drive. The proposed model illustrates the converter behavior when converting the input vibration energy into electricity [38]. A Supplementary Martial which summarizes the basic concept of the converter operation is provided. The output from the converter proposed model was the gained energy. The converter resonant frequency, which the converter spring is responsible for, was included in the calculation of the converter output power [38]. In this work, both the converter comb drive and spring design are considered. In this section, firstly, the most suitable spring configuration for the electrostatic MEMS converter operation is qualitatively determined. Then, the design of such a suitable configuration is illustrated.

### 2.1. The Common Geometries of MEMS Spring

**Figure 1:**
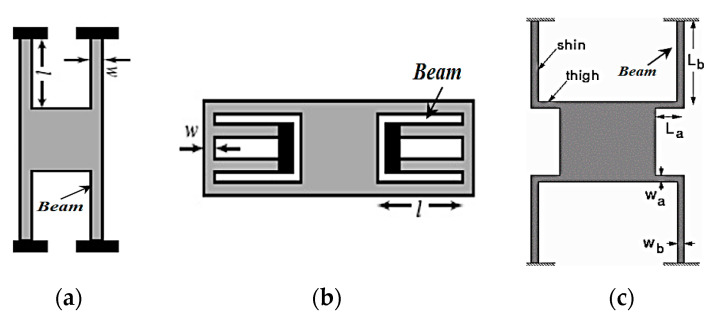
The common MEMS spring geometries: (**a**) fixed–fixed flexures, (**b**) folded flexures, and (**c**) crab leg flexures.

In this subsection, the commonly used MEMS spring geometries are presented. A comparison between such geometries is carried out to determine the most suitable type for the in-plane gap-closing electrostatic MEMS converter operation [39]. Figure 1a–c demonstrates the common MEMS spring geometries which are fixed–fixed, folded, and crab leg flexures.

Referring to Figure 1a, the fixed–fixed flexure spring geometry has an extensional axial stress in its beam. Thus, the spring constant of this geometry is very stiff and non-linear. Thus, it will not support the converter motion in the required *x* direction [39,40]. Concerning the folded flexure, referring to Figure 1b, it has a good compromise of linear behavior to an extent in the *y* direction. In addition, it has an added stiffness in the *x* direction. Therefore, it will resist the converter motion in the *x* direction [39,40]. In Figure 1c, the crab leg flexure is a modified version of the fixed–fixed flexure configuration. It has an added thigh to the beam which is used to minimize the peak stress [41]. This configuration is used to reduce the extensional axial forces of the beam [40]. Moreover, the crab leg flexure offers the required symmetry which is suitable for the in-plane gap-closing converter to function [42]. The most suitable spring type for the in-plane gap-closing electrostatic MEMS converter is the crab leg flexure, as it supports the motion in the desired *x* direction which is the required direction of motion of the converter [39,40,41,42]. Moreover, it reduces the extensional axial stress and provides better symmetry. Thus, the converter becomes safe from fracture during its operation [39,40,41,42].

### 2.2. Crab Leg Spring Design

The design of the crab leg spring, which is the most suitable MEMS spring geometry for the in-plane gap-closing electrostatic MEMS converter operation, aims to adjust the converter resonant frequency to be tuned to the desired frequency of the required application [38]. In this subsection, the design of the crab leg spring is illustrated. Firstly, the main governing equations which are used to design the crab leg flexure are presented. Then, the technological parameters of such spring are analytically determined. The spring constant (*k*) is determined by Equation (1) [39]:

(1) $$f_{0}=\frac{1}{2\pi }\sqrt{\frac{k}{m}}$$

where m is the shuttle mass, as given by Equation (2):

(2) $$m=\rho tA_{movable}$$

where *ρ* (= 2.33 g/cm^3^) is the density of poly-Si, which is the material utilized for fabricating the spring, and *t* is the converter thickness. *A_movable_* is the shuttle mass area and equals *L_m_* (*W_m_ +* 2 *L_f_*), *L_m_* and *W_m_* are the shuttle mass length and width, respectively, and *L_f_* is the finger length. The values of *L_m_, W_m_*, and *L_f_* are 1 cm, 0.3 cm, and 512 µm, respectively [38]. By using Equations (1) and (2), *k* is calculated to be 11.55 × 10^3^ N/m. Recalling the equations of the crab leg [39], the spring constants in the *x* and *y* directions, *k_x_*, and *k_y_*, are given by Equations (3), and (4), respectively:

(3) $$k_{x}=\frac{EtW_{b}^{3}}{L_{b}^{3}}$$

(4) $$k_{y}=\frac{EtW_{a}^{3}}{L_{a}^{3}}$$

Now, referring to Figure 1c, the spring dimensions need to be evaluated. These dimensions are beam length (*L_b_*), thigh length (*L_a_*), and the widths of the beam and thigh which are (*W_b_*, and *W_a_*), respectively. *L_b_* is calculated by using the maximum spring deflection in the following equation:

(5) $$Z_{\max}=\frac{2\sigma L_{b}^{2}}{3n_{s}tE}$$

*Z_max_* is the maximum displacement. The values of *Z_max_* and *t* are 6.75 µm and 500 µm, respectively [38]. *σ* and *n_s_* are the fracture stress of polysilicon and safety factors and equal 7 GPa, and 1.8, respectively [43]. Substituting Equation (5), *L_b_* is evaluated to be 0.7 mm. The spring constant that is calculated using Equation (1) is assumed to be in the desired direction of motion of the in-plane gap-closing converter (*k_x_*). Recalling Equations (3) and (4), *k_y_* must be larger than *k_x_* to avoid the converter motion in the undesired Y direction [44]. Thus, *k_y_*/*k_x_* is taken to be 500. In addition, for the homogeneity of the spring, we assume that *W_a_* = *W_b_* = *W_s_*. Combining Equations (3) and (4), *L_a_* and *W_s_* are evaluated to be 88 µm and 23 µm, respectively. All calculated parameters along with their respective definitions are summarized in Table 1.

## 3. Calibration of COMSOL MultiPhysics 5.4

In this section, the simulation of the electrostatic MEMS converter is carried out by using COMSOL Multiphysics 5.4. The simulator is calibrated by using the technological parameters of the electrostatic MEMS converter case study found in [38]. The converter performance is verified by achieving the simulation results of five main performance indicators which are electric potential and the electric field distribution, the converter fingers displacement and the stress analysis due to the input vibration signal, and the converter output power (*P_out_*) is simulated at different input voltage (*V_ip_*).

### 3.1. The Electric Potential and Electric Field Distributions

**Figure 2:**
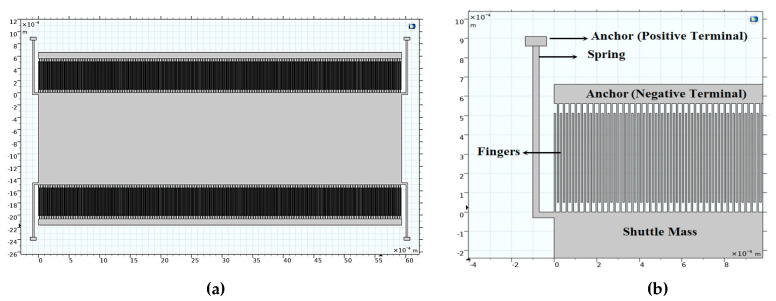
(**a**) The 2D converter structure and (**b**) a focus of the structure showing a clear view.

**Figure 3:**
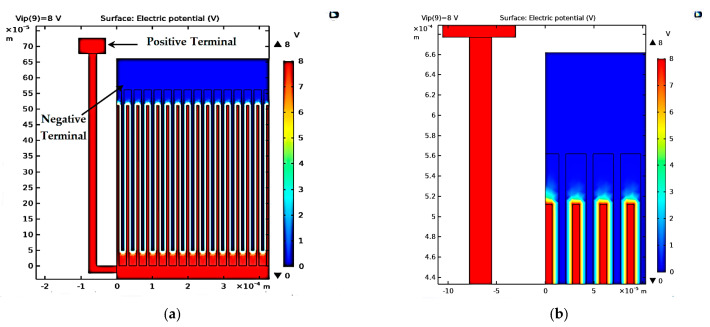
The converter electric potential distribution’s (**a**) overall structure and (**b**) a focus showing a clearer view.

**Figure 4:**
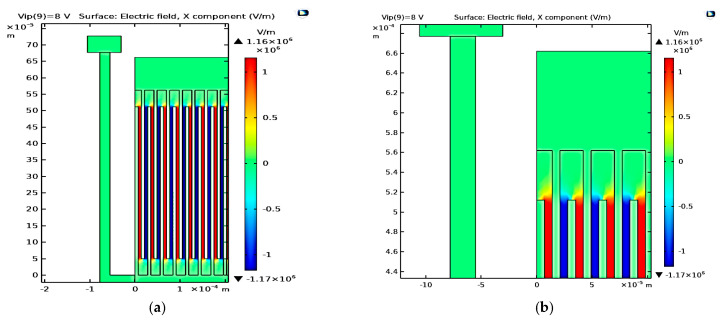
The electric field distribution’s (**a**) overall structure and (**b**) a focus showing a clearer view.

First, Figure 2a demonstrates the 2D converter structure for 0.35 µm CMOS technology by using COMSOL Multiphysics 5.4. Figure 2b shows a part of the structure which clarifies its details. Upon simulating the structure given the appropriate boundary conditions, the electric potential distribution is demonstrated in Figure 3a,b. The voltage distribution changes from 0 V to *V_max_,* which is 8 V for the 0.35 µm design [38]. Furthermore, the electric field distribution simulation is performed to check the safe design of the converter concerning the maximum electric field that exists between the converter fingers. Figure 4a,b show the simulation results of the electric field distribution with a focus on Figure 4b, which clarifies the results. It is clear that the value of the maximum electric field is 1.16 × 10^6^ V/m, which is less than half of the air breakdown electric field, which is 1.5 × 10^6^ V/m [38]. This result guarantees that the converter design is safe.

### 3.2. The Converter Displacement due to the Input Vibration Signal

**Figure 5:**
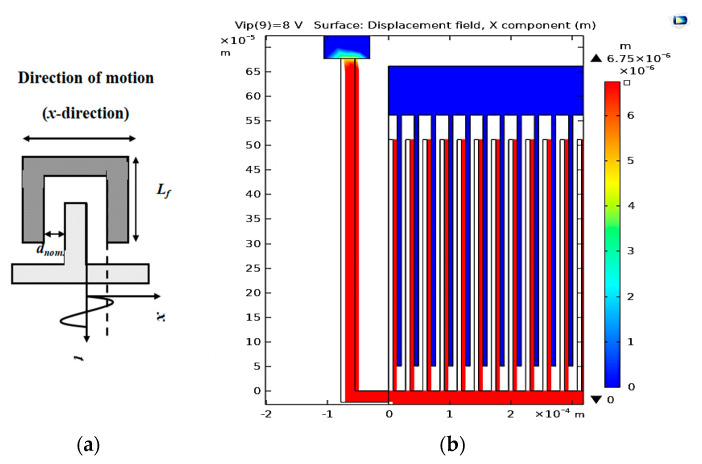
(**a**) The two finger representation of the converter and (**b**) the converter’s maximum displacement.

In this subsection, the input vibration signal of the vibration source is applied to the converter. Thus, the maximum displacement of the converter can be measured. As shown in Figure 5a, the nominal gap between converter fingers at rest position (*d_nom_*), at which the minimum capacitance of the converter occurs, is 7 µm. Additionally, the minimum distance (*d_min_*) at which the maximum capacitance of the converter occurs is 0.25 µm. Thus, the maximum displacement of the converter fingers must be *d_nom_*–*d_min_* = 6.75 µm. Figure 5b shows the simulation results of the converter’s maximum displacement. It is equal to 6.75 µm, which quantitatively verifies the analytically calculated value based on Figure 5a. This value satisfies the proper operation of the converter.

### 3.3. The Stress Analysis for the Converter due to the Input Vibration Signal

**Figure 6:**
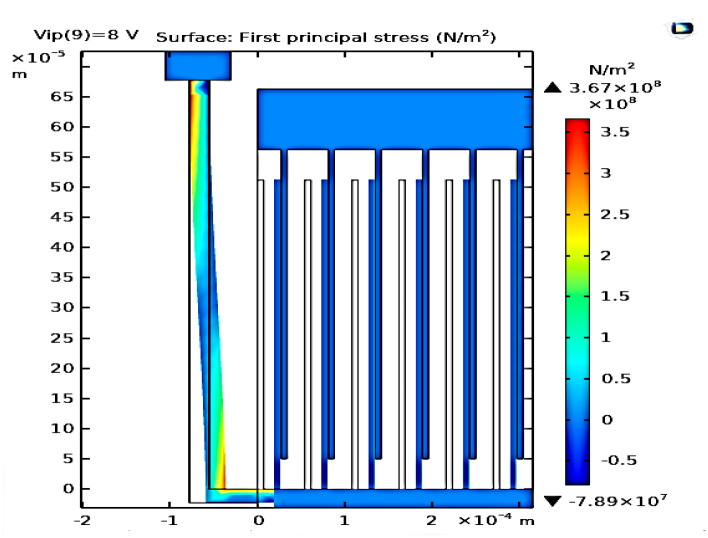
The converter stress analysis is due to the input vibration signal.

In this subsection, stress analysis for the converter due to the input vibration signal is investigated. Such simulation is important to guarantee that the design is safe against fracture. Figure 6 shows the simulation results of the converter stress. It is obvious that the maximum stress is 3.67 × 10^8^ N/m^2^, which is 0.367 Gpa. This value is smaller than the fracture stress of the polysilicon, which is 7 GPa [43]. Thus, the design is safe against fracture.

### 3.4. The Converter Outputs Power at Different V_ip_

**Figure 7:**
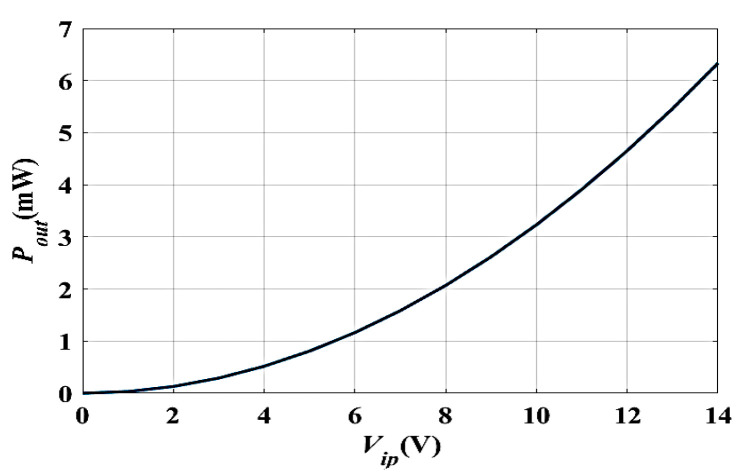
The simulation result of *P_out_* vs. *V_ip_*.

In this subsection, the converter output power (*P_out_*) is simulated by sweeping the input voltage (*V_ip_*). The output power *P_out_* is directly proportional to the square of *V_ip_,* as indicated in Ref. [38]. Therefore, the maximum value of *P_out_* (*P_outmax_*) has to occur at *V_ip_* equal to the maximum voltage of the system, which is 8 V for the used case study. Additionally, *P_out_* is expected to increase with the increase in *V_ip_*. Figure 7 shows the simulation results of *P_out_* vs. *V_ip_* for sweeping *V_ip_* from 0 V to 14 V. It is obvious that the maximum value of *P_out_*, which is 2.1 mW, occurs at *V_max_*, which is 8 V, which is in agreement with the gained value from the converter model and the analytically calculated values, which were 2.2 mW and 2.3 mW, respectively [38]. Based on the previous calculations and results, the COMSOL tool is calibrated to be used for optimizing the electrostatic MEMS converter structure to enhance its performance.

## 4. Qualitative Analysis of the Three Performance-Enhancing Techniques

In this section, the performance enhancement of the converter is investigated to boost its output power. Three effective performance enhancement techniques are applied to achieve such an objective. Firstly, the converter output power is boosted by scaling up the technology of the converter CMOS circuit, the power conditioning, and power control circuits, from 0.35 µm to 0.6 µm CMOS technology. Secondly, the converter output power increases by optimizing its technological parameters, namely the converter thickness and converter finger width and length. Finally, the converter structure is optimized to maximize its finger length.

Concerning the first technique, the technology of the converter circuit is scaled up from 0.35 µm to 0.6 µm CMOS technology. Such scaling up has the following advantages. Firstly, it increases the main effective system constraint, *V_max_*. As the converter output power is directly proportional to the square of *V_max_* [38]; so, scaling up the technology is expected to effectively enhance the converter output power. Moreover, when *V_max_* increases, the nominal distance (*d_nom_*) between the converters’ fingers increases, resulting in decreasing the number of fingers. Additionally, the aspect ratio (AR) of the deep reactive ion etching (DRIE) fabrication process decreases. Thus, the converter fabrication cost decreases.

To qualitatively calculate the maximum voltage (*V_max_*) of 0.6 µm CMOS technology, its breakdown voltage (*V_BD_*) must be specified. From the 0.6 µm CMOS technology file [45], *V_BD_* is 62 V. For a safe design of the power switches found in the system power condition circuit, *V_max_* is assumed to be approximately equal to half of *V_BD_* [38]; thus, *V_max_* is assumed to equal 30 V. Based on the value of *V_max_*, the nominal distance between the converter fingers (*d_nom._*) must be calculated to satisfy the safe design. The nominal distance is determined using Equation (6):

*E_max_ = V_max_/d_nom._* (6)

*E_max_* is the maximum electric field that occurs between the converter fingers. For a safe design, it is assumed to be half of the breakdown field of air. Thus, *E* equals 1.5 × 10^6^ V/m [46], from which *d_nom_* is calculated to be 20 µm.

The second applied performance enhancement technique is the optimization of the converter technological parameters which are the finger length (*L_f_*), finger width (*W_f_*), and the converter thickness (*t*). Equations (7)–(9) represent the converter number of fingers (*N_g_*), maximum capacitance (*C_max_*), and minimum capacitance (*C_min_*).

(7) $$N_{g}=\frac{L_{m}}{\left(W_{f}+2\times d_{nom.}\right)}$$

(8) $$C_{max}=\frac{4N_{g}\varepsilon_{0}\varepsilon_{r}L_{f}td_{nom.}}{\left(d_{nom.}^{2}-Z_{\max}^{2}\right)}$$

(9) $$C_{\min\;}=\frac{4N_{g}\varepsilon_{0}\varepsilon_{r}L_{f}t}{d_{nom.}}$$

Based on the above equations, to increase *P_out_*, the converter capacitances must increase [38]. To increase the capacitances the converter finger length and thickness, the number of fingers must increase. Concerning the finger width, using Equation (7) to increase the number of fingers, the finger width must decrease. In the optimization of converter technological parameters there are essential technological limitations and restrictions. Such limitations affect the optimum values of each technological parameter. In the Section 5, these limitations will be illustrated.

The third applied performance enhancement technique is the optimization of the converter structure. Such optimization aims to overcome the converter wasted shuttle mass area, which will be illustrated in the coming sections.

## 5. Enhancing the Converter Performance Using COMSOL Simulations

In this section, the electrostatic MEMS converter performance based on the three applied enhancement techniques is simulated using COMSOL Multiphysics 5.4.

### 5.1. Scaling up the Technology

**Figure 8:**
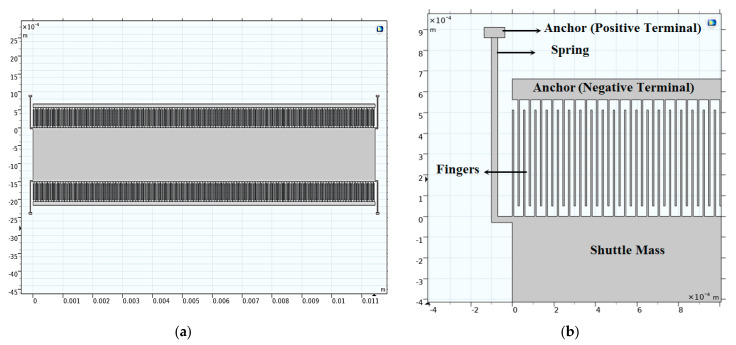
(**a**) The 2D converter structure and (**b**) a focus of the structure showing a clear view.

As mentioned herein, the converter out power becomes 4.5 mW when scaling up to 0.6 µm CMOS technology. This value is double the output power in the case of 0.35 µm CMOS technology, which was 2.1 mW. The converter performance based on the technology scaling is simulated to ensure the proper operation of the converter under investigation. The electric potential and the electric field distributions, the converter displacement, and the stress analysis due to the input vibration signal, and the converter output power at different *V_ip_* is simulated. Figure 8a demonstrates the 2D converter structure for 0.35 µm CMOS technology by using COMSOL Multiphysics 5.4. Figure 8b shows a part of the structure which clarifies its details.

**Figure 9:**
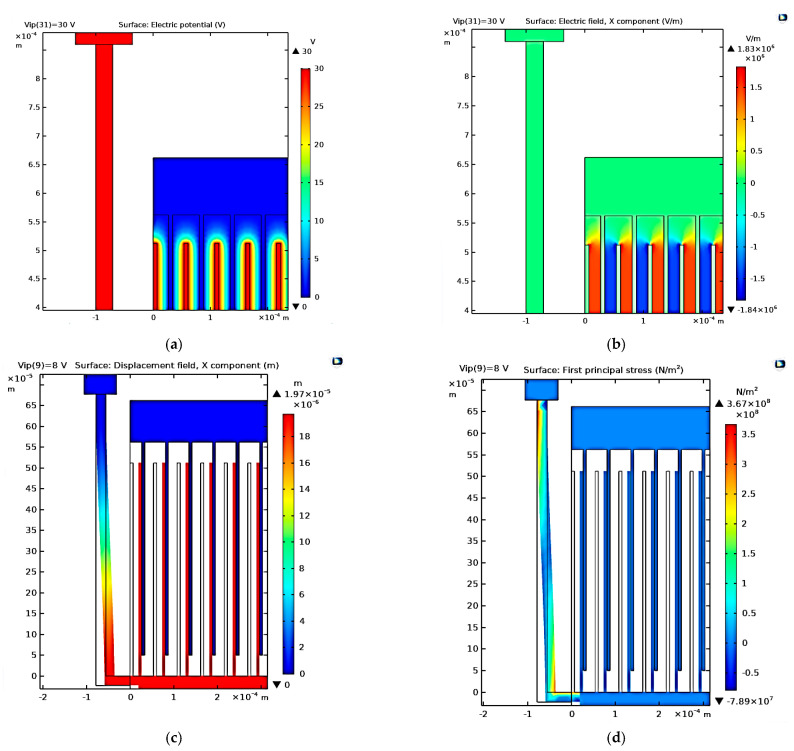

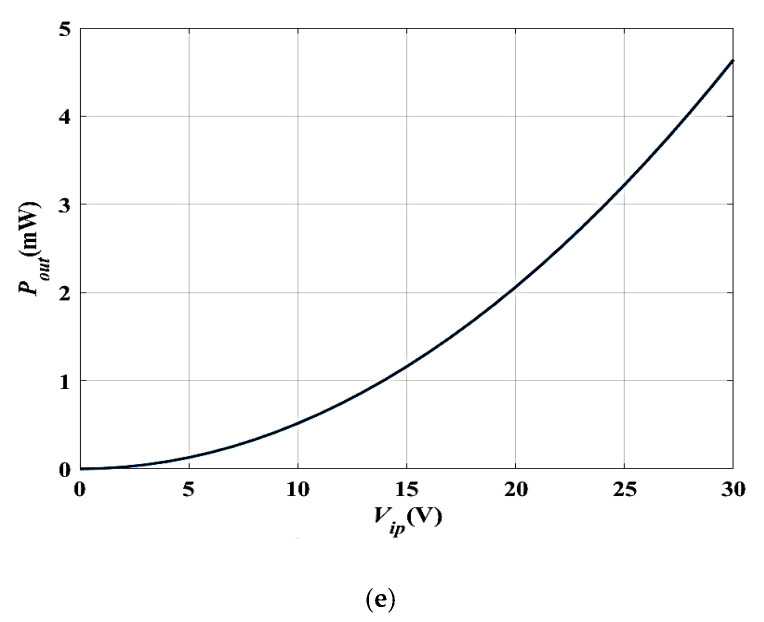
(**a**) Electric potential distribution, (**b**) electric field distribution, (**c**) converter displacement due to the input vibration signal, (**d**), stress analysis, and (**e**) *P**_out_*** at different values of *V_ip_*.

Figure 9a–e show the simulation results of the electric potential distribution, electric field distribution, the converter displacement due to the input vibration signal, the stress analysis, and the converter output power at different values of *V_ip_*. In Figure 9a, it is clear that the maximum voltage, *V_max_*, is 30 V, which numerically verifies the qualitative value in Section 4. Figure 9b displays the maximum electric field, which is 1.83 × 10^6^ V/m. It is less than half of the air breakdown electric field, which is 1.5 × 10^6^ V/m [46]; thus, the design is safe. In Figure 9c, the maximum displacement is 19.7 µm, which agrees with the analytical value *d_mon._*–*d_min._* = 20 µm–0.25 µm = 19.75 µm in Section 4. Further, regarding Figure 9d, the value of the maximum stress is found to be 0.36 Gpa, which is again smaller than the fracture stress of the polysilicon. Thus, the converter design is safe against fracture. Finally, concerning Figure 9e, it is obvious that the value of the simulated *P_outmax_* at *V_max_* is 4.5 mW, which is double the value found for 0.35 µm CMOS technology.

### 5.2. Technological Parameters Optimization

**Figure 10:**
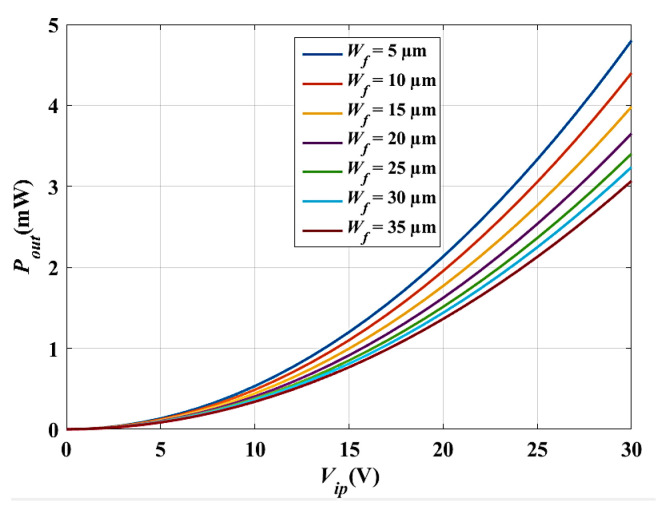
*P_out_* versus *V_ip_* at different *W_f_*.

Here, the converter performance is enhanced by optimizing its technological parameters which are the converter thickness (*t*), finger width (*W_f_*), and finger length (*L_f_*). Concerning the device thickness (*t*), recalling Equations (7)–(9) of *N_g_*, *C_max_*, and *C_min_*, it is obvious that *C_max_* and *C_min_* increase by increasing the device thickness (*t*). As a result, the output power of the converter increases. In this work, *t* is selected to be 500 µm, as it is the standard device thickness for SOI technology [19,47]. Concerning the converter finger width (*W_f_*), it is clear using Equation (7) that *N_g_* increases by decreasing *W_f_*. Thus, *C_max_, C_min_*, and *P_out_* will increase. The converter output power is calculated and simulated for different values of *W_f_*. The optimum value of *W_f_* that achieves the highest output power is found to be 5 µm. Table 2 represents the calculated and simulated values of *P_out_* at different *W_f_*. Figure 10 shows *P_out_* versus *V_ip_* at different *W_f_*. It is obvious that *P_out_* increases by decreasing *W_f_.* In this work, the value *W_f_* is set to 10 µm to guarantee the rigidity of the structure.

**Figure 11:**
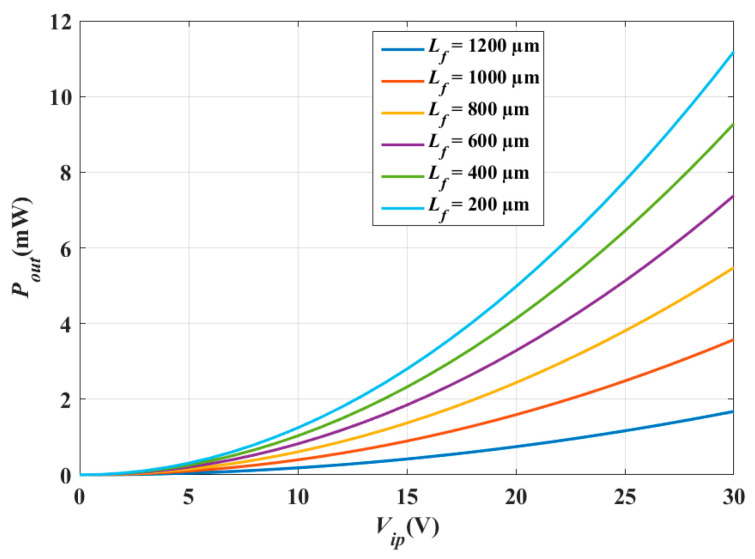
*P_out_* versus *V_ip_* at different *L_f_*.

**Figure 12:**
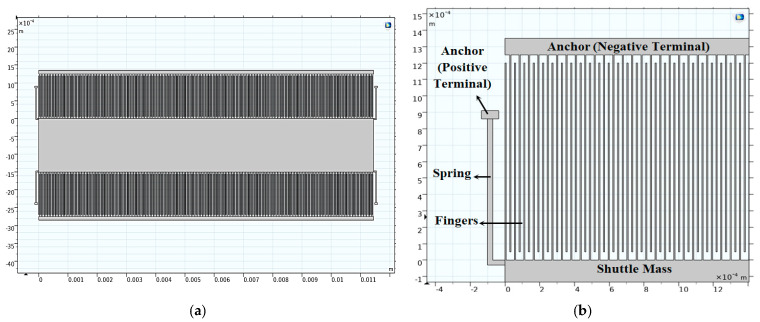
(**a**) The 2D optimized converter structure and (**b**) a focus of the structure showing a clear view.

For the converter finger length (*L_f_*), recalling Equations (8) and (9), it is obvious that *C_max_* and *C_min_* increase by increasing *L_f_.* Thus, *P_out_* also increases. Table 3 represents the calculated and simulated values of *P_out_*, at different values of *L_f_* [48]. Furthermore, Figure 11 shows *P_out_* versus *V_ip_* at different *L_f_.* As is depicted, *P_out_* increases by increasing *L_f_*. In this work, the value *L_f_* is set to 1200 µm, achieving the optimum *P_out_.* Figure 12a demonstrates the 2D converter structure for 0.35 µm CMOS technology by using COMSOL Multiphysics 5.4. Figure 12b shows a part of the structure which clarifies its details.

**Figure 13:**
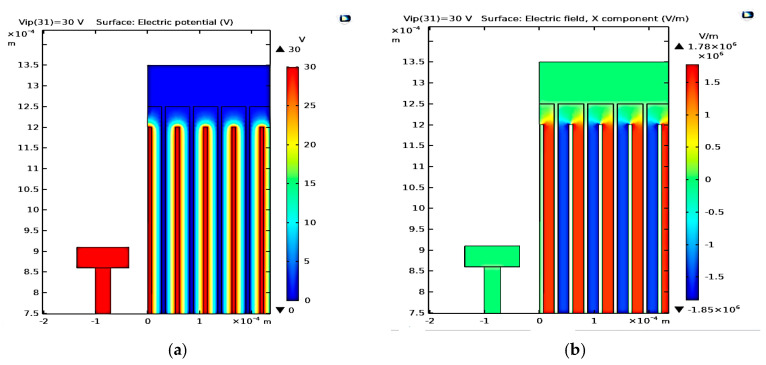

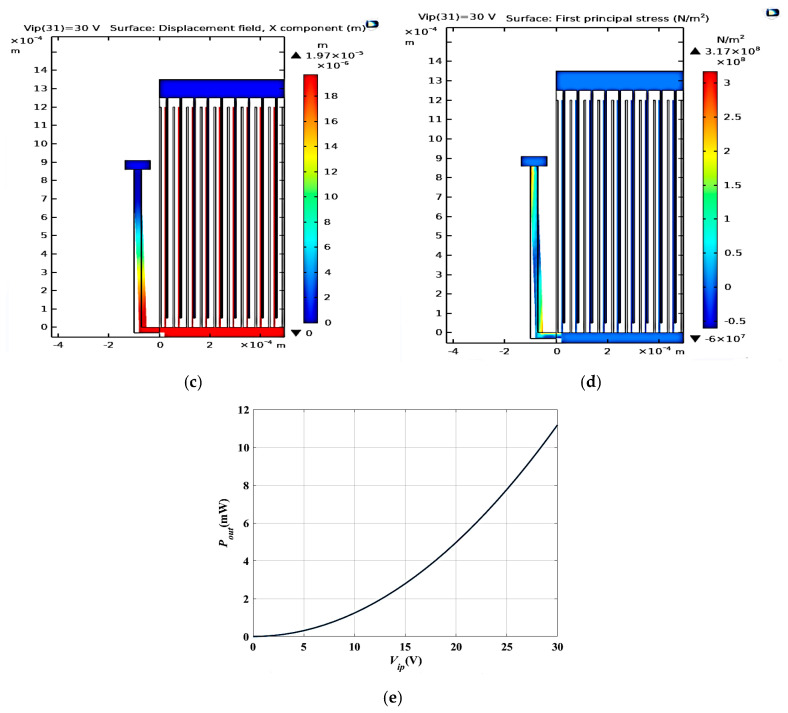
(**a**) Electric potential distribution, (**b**) electric field distribution, (**c**) converter displacement due to the input vibration signal, (**d**), stress analysis, and (**e**) *P_out_* at different values.

Figure 13a–e show the simulation results of the optimized converter; the maximum voltage is 30 V, as indicated in Figure 13a, which agrees with the analytical value. In addition, the maximum electric field is 1.78 × 10^6^ V/m according to Figure 13b, confirming the safety design criterion. Furthermore, the maximum displacement is 19.7 µm, extracted from Figure 13c, which agrees with the analytically calculated value. Furthermore, as can be inferred from Figure 13d, the value of the maximum stress is 0.317 Gpa, revealing the safety against fracture. Finally, *P_outmax_* is 11.2 mW according to Figure 13e, which illustrates the variation of *P_out_* versus *V_ip_*. This simulated value also agrees with the analytically calculated value.

Remarkably, the most effective technological parameter which significantly influences the converter performance is *L_f_*. Referring to Figure 12, it is not recommended that *L_f_* exceeds 1200 µm [19,47,48]. From fabrication visibility, the converter finger becomes so long that it can be easily broken. Thus, if *L_f_* exceeds 1200 µm, the converter will become fragile. For the converter structure to be optimized, one has to maximize the converter finger length without being fragile.

### 5.3. The Electrostatic MEMS Converter Structure Optimization

**Figure 14:**
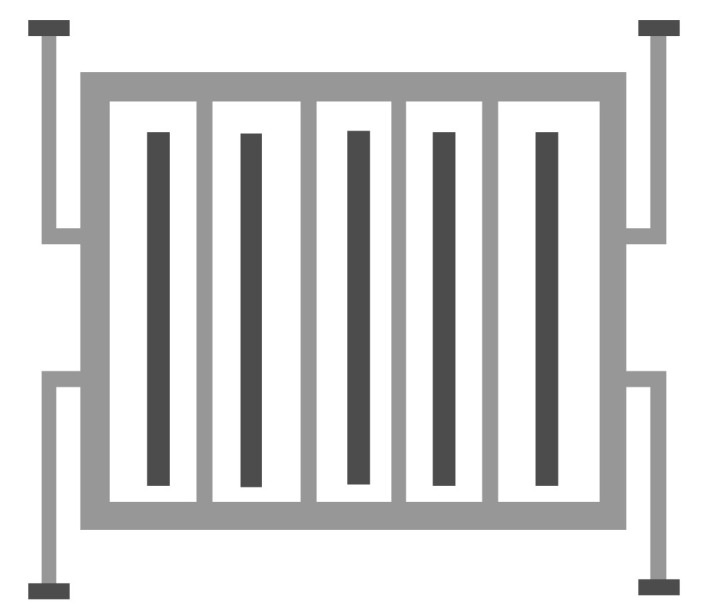
Proposed electrostatic MEMS converter.

**Figure 15:**
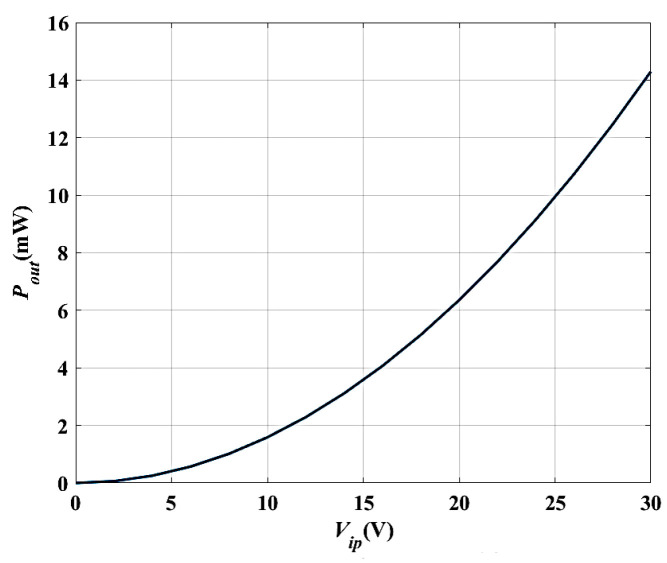
Simulation results of the proposed converter output power with the input voltage.

Next, the converter performance is enhanced by modifying and optimizing its structure. Figure 14 demonstrates the proposed converter structure which has the following advantages. First, it makes the best use of the wasted shuttle mass area by evacuating its center. Secondly, the finger length increases; thus, the output power also increases. Finally, the converter becomes ridged because of the continuous fingers which are anchored from both sides. Figure 15 shows the simulation results of the proposed converter output power with the input voltage. As depicted in the figure, the output power becomes 14.29 mW. Thus, the proposed structure enhances the converter output power further by 3 mW.

As a comparison with the cited work in [30], multi-vibrational mode electrostatic energy harvesters have been designed. An output of 2.96 µW at an input vibration frequency of 1.272 kHz has been obtained [30]. Further, a symmetric comb electrode has been used at an input vibration frequency of 125 Hz and an output power of 70 µW has been provided [31]. An electret vibration energy harvester was used which provides an output power of 495µW at an input vibration frequency of 1.2 kHz [32]. Concerning Ref. [47], gap-closing inter-digitated electrodes electrostatic MEMS vibration energy harvesters were used at an input vibration frequency of 120 Hz and gave 3.13 µW of output power. A 2DOF e-VEH MEMS device with impact-induced nonlinearity was utilized where operation at an input vibration frequency of 731 Hz was employed [49]. Such type gives an output power of 14 µW. Moreover, in Ref. [50], a batch-fabricated, low-frequency, and wideband MEMS electrostatic vibration energy harvester has been used at an input vibration frequency of 428 Hz and an output power of 6.6 µW was recorded. Finally, an out-of-plane electret-based vibrational energy harvester was introduced at an input vibration frequency of 95 Hz giving an output power of 0.95 μW [51].

Table 4, summarizes the output power of the proposed electrostatic MEMS converter in comparison with cited studies. It is obvious that the proposed converter using 0.6 µm CMOS technology is promising, as it achieves a high 14.29 mW output power. After exploring the design space of all kinds of parameters, there is an important intrinsic tradeoff and challenges for the proposed electrostatic MEMS converter. Such challenges concern the reduction in the converter area which will effectively reduce its fabrication cost. Previously, there was a tradeoff between reducing the converter area to reduce the fabrication cost which greatly degrades the converter performance, mainly its output power. In our promising proposed converter, a proposed solution to such an issue is introduced. The reason is that our converter generates a relatively high output power when compared with the recent research. Thus, based on the application, its area can be reduced while maintaining the satisfactory output power which was not applied before. In addition, there was a main challenge faced by the converter which is its fragility after fabrication. This is because the finger length must be large to achieve higher output power. Thus, the finger can be easily broken. However, our promising proposed converter is less fragile and more ridged. The reason is that the converter fingers are attached from two sides in a structure that looks like a net, as shown in Figure 4. Thus, it will not suffer from being broken.

## 6. Conclusions

In this work, the electrostatic MEMS converter performance is enhanced by using three effective techniques. Firstly, the converter output power is boosted by scaling up the technology of its accompanied CMOS circuit, the power conditioning and power controlling circuits, from 0.35 µm to 0.6 µm CMOS technology. The maximum voltage of the energy harvesting system, *V_max_*, is the most effective system constraint that increases from 8 V to 30 V. Thus, the converter output power is doubled from 2.1 mW to 4.5 mW. Secondly, the converter output power increases by optimizing its technological parameters, the converter thickness and the converter finger width and length. The optimum values of the converter parameters which achieve the optimum output power are *t* equals 500 µm, *W_f_* is selected to be 10 µm, and *L_f_* equals 1200 µm. Thus, the converter output power increased from 4.5 mW to 11.2 mW. From such optimization, *L_f_* is found to be the most effective technological parameter which affects the converter performance. It is recommended to maximize *L_f_;* however, this objective cannot be achieved with the traditional electrostatic MEMS converter as long as *L_f_* increases and the converter becomes fragile. The third optimization technique aims to maximize the converter finger length by optimizing the converter structure. A proposed structure aims to overcome the wasted area of the shuttle mass and maximize *L_f_* by anchoring it from both sides. Thus, the converter becomes more ridged. The proposed structure enhances the converter output power from 11.2 to 14.29 mW. All the simulations are carried out by using COMSOL Multiphysics 5.4. In future work, the bandwidth broadening of the electrostatic MEMS converter will be investigated. Thus, the converter will resist performance degradation due to any shift in its resonant frequency.

## Figures and Tables

**Table 1:** A summary of the main technological design parameters.

Parameter	Definition	Value (unit)
*t*	Converter thickness	500 µm
*L_m_*	Shuttle mass length	1 cm
*W_m_*	Shuttle mass width	0.3 cm
*L_f_*	Finger length	512 µm
*L_b_*	Beam length	0.7 mm
*L_a_*	Thigh length	3.2 mW
*W_s_*	Spring width	3.06 mW

**Table 2:** *P_out_* at different *W_f_*.

*W_f_*	*P_out_* Calculated	*P_out_* Simulated
5 µm	5.3 mW	4.5 mW
10 µm	5 mW	4.3 mW
15 µm	4.5 mW	4 mW
20 µm	4.1 mW	3.6 mW
25 µm	3.8 mW	3.4 mW
30 µm	3.55 mW	3.2 mW
35 µm	3.3 mW	3.06 mW

**Table 3:** *P_out_* at different *L_f_*.

*L_f_*	*P_out_* Calculated	*P_out_* Simulated
200 µm	2 mW	1.96 mW
400 µm	4.1 mW	3.59 mW
600 µm	6.19 mW	5.5 mW
800 µm	8.26 mW	7.4 mW
1000 µm	10.3 mW	9.3 mW
1200 µm	12.39 mW	11.2 mW

**Table 4:** Performance parameters comparison of different electrostatic harvesters.

Work	Converter Type	Frequency (kHz)	Output Power (mW)
[30]	Multi-vibrational mode	1.272	0.00296
[31]	Symmetric comb electrode	0.125	0.070
[32]	Electret vibration energy harvester	1.2	0.495
[47]	Gap-closing electrostatic MEMS vibration energy harvester	0.12	0.00313
[49]	2DOF e-VEH MEMS device with impact-induced nonlinearity	0.731	0.014
[50]	Batch-fabricated, low-frequency, and wideband MEMS electrostatic vibration energy harvester	0.428	0.0066
[51]	Out-of-plane electret-based vibrational energy harvester	0.95	0.00095
This work	In-plane gap-closing converter using 0.35 µm CMOS technology	2.5	2.1
This work	In-plane gap-closing converter using 0.6 µm CMOS technology	2.5	4.5
This work	In-plane gap-closing proposed converter using 0.6 µm CMOS technology	2.5	14.29

## Data Availability

No new data were created or analyzed in this study. Data sharing is not applicable to this article.

## Supplementary Materials

https://www.mdpi.com/article/10.3390/mi14020485/s1.
