# Supplementary material for: Boosting the Electrostatic MEMS Converter Output Power by Applying Three Effective Performance-Enhancing Techniques
Source: Micromachines (Basel). 2023 Feb 19;14(2):485. doi: 10.3390/mi14020485 (PMC9964943; doi:10.3390/mi14020485)
Supplement: Supplementary file 1 [file micromachines-14-00485-s001.zip › micromachines-2177934-supplementary.pdf]

Supplementary Materials

Boosting the Electrostatic MEMS Converter Output Power by Applying Three Effective Performance Enhancing Techniques

Mona S. Salem, A. Zekry, M. Abouelatta, A. Shaker, Marwa S. Salem, Christian Gontrand and Ahmed Saeed

S1. Electrostatic MEMS Converter and Main Parameters

Figure S1.a demonstrates the conventional in-plane gap-closing electrostatic MEMS converter, while Figure S1.b shows the details of its main technological parameters, respectively [S1]. The converter comb drive technological parameters are identified in Table S1. The converter comb drive has two main capacitances according to its fingers position with respect to its fixed comb and anchors, which are minimum capacitance (C_{min}) and maximum capacitance (C_{max}), expressed by Equations (1) and (2), respectively.

$$C_{\min} = \frac{4N_g \varepsilon_0 \varepsilon_r L_f t}{d_{nom.}} \tag{1}$$

$$C_{\max} = \frac{4N_g \varepsilon_0 \varepsilon_r L_f t d_{nom.}}{\left(d_{nom.}^2 - Z_{\max}^2\right)} \tag{2}$$

where: N_g is the number of fixed/movable fingers; d_{nom} is the nominal gap between fingers at rest position, and Z_{max} is the maximum deflection of the converter springs from their rest position [S1].

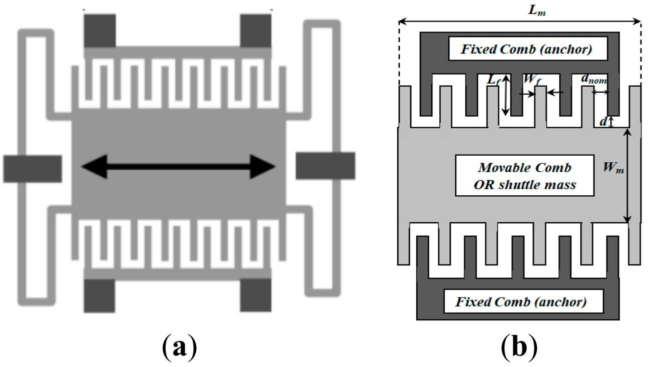

Figure S1. (**a**) The conventional gap-closing electrostatic MEMS converter and (**b**) A detailed description of the converter comb drive parameters [S1].

Table S1. Main technological parameters of the conventional gap-closing electrostatic MEMS converter comb drive for the case study.

Symbol	Technological Parameter	Value
L_f	Finger Length	512 µm
W_f	Finger Width	7 µm
$d_{nom.}$	Nominal Gap between Fingers at rest position	7 µm
d	Dielectric Distance	50 µm
L_m	Shuttle Mass Length	1 cm
W_m	Shuttle Mass Width	0.3 cm

$L_{terminal}$	Positive Terminal Length	100 µm
$W_{terminal}$	Positive Terminal Width	100 µm
N_g	Number of fixed or movable fingers	476
t	Device thickness	500 µm

S2. Main Governing Equations of the Electrostatic MEMS Converter

In this section, the main analytical equations which govern the converter performance are illustrated. Based on Figure S1, during the input vibration, the converter movable combs and spring move in the lateral direction. The capacitance changes due to the change of the dielectric gap between the combs drive fingers. The converter output power is given by Equation (1) [S1, S2].

$$P_{out} = 2 f_0 E \quad (1)$$

where P_{out} is the output power of the converter, E_{useful} is the useful energy per cycle and f_0 is the driving frequency of the input vibration source, which is also the converter resonant frequency. The converter is designed to operate based on the source of the input vibration taken from a gas turbine. Thus, the converter can be used in several different modes in critical industries such as power generation, oil and gas, process plants, aviation, as well domestic and smaller related applications [S3, S4].

After analytical derivation which is illustrated in details in our previous research work [S5], the converter output power is given by Equation (2):

$$P_{out} = C_{\min} V_{\max}^2 (1-\alpha) f_0 \quad (2)$$

where $\alpha = C_{min}/C_{max}$, V_{max} is the maximum voltage, which is one of the basic key factors of the vibration energy harvesting system.

References

[S1] Tousif, S.R. MEMS Wideband Energy Harvesting Using Nonlinear Springs and Mechanical Stoppers. Ph.D. Thesis, The University of Texas, Arlington, TX, USA, 2019.

[S2] Roundy, S.J. Energy Scavenging for Wireless Sensor Nodes with a Focus on Vibration to Electricity Conversion. Ph.D. Thesis, University of California, Berkeley, CA, USA, 2003.

[S3] Mur Miranda, J.O. Electrostatic Vibration-to-Electric Energy Conversion. Ph.D. Thesis, Massachusetts Institute of Technology, Cambridge, UK, 2004

[S4] Isaiah, T.G.; Dabbashi, S.; Bosak, D.; Sampath, S.; Di Lorenzo, G.; Pilidis, P. Life analysis of industrial gas turbines used as a back-up to renewable energy sources. *Procedia CIRP* **2015**, *38*, 239–244

[S5] Salem, M. S., Zekry, A., Abouelatta, M., Shaker, A., & Salem, M. S. Validation and Evaluation of a Behavioral Circuit Model of an Enhanced Electrostatic MEMS Converter. Micromachines 2022, 13(6), 868, https://doi.org/10.3390/mi13060868.
